# Identifying mounting behaviour in boars using deep learning-based instance segmentation and binary classification - a pilot study

**DOI:** 10.3389/fbinf.2026.1869902

**Published:** 2026-09-04

**Authors:** Harry Aricibasi, Jessica Bode, Renée Bergeron, Dan Tulpan

**Affiliations:** Department of Animal Biosciences, University of Guelph, Guelph, ON, Canada

**Keywords:** boars, classification, computer vision, deep learning, mounting, segmentation, swine

## Abstract

**Introduction:**

Mounting behaviour in group-housed pigs poses significant welfare and productivity challenges, often resulting in injuries and stress. To address this issue, we developed a novel computer vision-based approach for the automatic detection of mounting behaviour in pigs.

**Methods:**

The pilot study was conducted on eight boars, aged 4–5 months and weighing between 60 and 90 kg, housed in two groups of four. Continuous video footage was collected over a 1-week period using an overhead camera, resulting in 766 2-s video clips. The proposed hybrid approach integrates Mask Region-based Convolutional Neural Network (Mask R-CNN) instance segmentation with a binary classifier. To evaluate the model’s performance under varying data conditions, the dataset was divided into training, validation, and testing sets using three data grouping scenarios: by frame, by clip, and by day.

**Results:**

Among the five evaluated binary classification algorithms, Support Vector Machine (SVM) was selected based on its superior performance. The hybrid system achieved a balanced accuracy and F1-score of 95% with the clip-based split, rising to 99% with the frame-based split and falling to 74% with the day-based split.

**Discussion:**

Under pilot conditions, the system offers a feasible proof-of-concept for continuous monitoring of mounting behaviour in group-housed pigs, operating at a processing rate of five frames per second. This capability offers strong potential for supporting early intervention and proactive welfare management, subject to future multi-site validation. Moving forward, future research will focus on integrating individual animal tracking and conducting larger-scale studies to further explore the system’s scalability and enhance its performance.

## 1 Introduction

For years, swine producers have routinely castrated male piglets shortly after birth. Recently, however, there has been growing advocacy to end this practice due to animal welfare concerns (Bonneau and Weiler, 2019). Moreover, one of the most common methods, surgical castration, is known to cause pain in male piglets during and after the procedure, as indicated by increased vocalization and biological stress markers, such as elevated heart rate and catecholamine levels in the blood (Hay et al., 2003). Keeping males intact also provides advantages, as intact males exhibit a 10%–15% improvement in feed efficiency and a 15% reduction in nitrogen excretion compared to castrates (Lundström et al., 2009). Raising boars instead of castrated males can therefore lower production costs and reduce the environmental impact of swine farming. Although there are strong arguments against castrating male pigs, raising intact males introduces its own set of economic and welfare challenges.

One such challenge in raising boars instead of castrated pigs is their heightened tendency for aggressive and sexual behaviours (Rydhmer et al., 2006). While both behaviours pose welfare concerns, this research specifically focuses on sexual behaviours, particularly mounting, since the automatic detection of aggression has been more extensively studied (Viazzi et al., 2014; Lee et al., 2016; Chen et al., 2019; Chen et al., 2020; Fraser et al., 2023). Another reason for prioritizing sexual behaviours like mounting is their increased prevalence in older boars. Studies show that younger boars exhibit more aggressive behaviours than females and castrated males, but as they age, their aggressive tendencies decline to levels comparable to other sexes, whereas mounting behaviour rises significantly (Clark and D’Eath, 2013; Lange et al., 2021).

Mounting is defined as one pig straddling the front or back of another pig using its front legs (Hintze et al., 2013). This behaviour negatively impacts welfare in group-housed pigs, leading to increased injuries, stress, and lameness (Rydhmer et al., 2006). Additionally, a review by Squires et al. (2020) emphasizes the negative economic effect of wounds and stress, which can reduce growth rates, increase medical costs, or, in severe cases, necessitate culling. Although mounting occurs more frequently in intact males than in castrated ones, research suggests that mounting behaviour is an individual trait rather than a random behaviour (Hintze et al., 2013). This means that while mounting is common within pens, it is typically carried out by a select few individuals repeatedly mounting other pigs in the pen. Therefore, continuous monitoring of pens to identify mounting behaviour could enable the isolation and separation of pigs exhibiting this behaviour, preventing further harm to themselves and others. While human observation, whether in person or through video review, might seem a straightforward solution, it presents several challenges, including observer bias, high labor costs, and the time-intensive nature of monitoring, especially in larger operations (Chen et al., 2021).

To address these challenges, we propose a hybrid system based on machine learning, deep learning and computer vision for the automatic detection of mounting behaviour. Machine learning is a branch of artificial intelligence, which simulates human learning through computing and enables computers to recognize patterns and make decisions by learning from data. Within the field of machine learning, deep learning is a set of techniques inspired by the structure of the human brain focused on solving complex challenges using large artificial neural networks. These networks process data through multiple layers, with each layer recognizing increasingly complex patterns. By identifying these patterns, deep learning models can accurately analyze and make predictions based on input data (Bengio, 2009). Computer vision is a branch of artificial intelligence focused on teaching computers to interpret and understand visual information. It allows machines to recognize and classify objects, detect patterns, and derive meaningful insights from images and videos, emulating, to some extent, how humans perceive and process visual data with their eyes and brains. Our approach integrates computer vision with deep learning to analyze videos and images for behavioural patterns recognition.

One critical technique that bridges deep learning and computer vision is image segmentation. Image segmentation refers to the process of dividing an image into meaningful regions or segments, often by assigning a label to every pixel in the image. This allows the model to differentiate between different objects or parts of a scene. There are three main types of image segmentation: semantic segmentation, instance segmentation and panoptic segmentation. Semantic segmentation classifies each pixel into a category (e.g., pig vs. background) without distinguishing between individual objects from the same category. Instance segmentation not only classifies pixels but also separates distinct instances of the same object (e.g., distinguishing between individual pigs in a group). Panoptic segmentation combines semantic and instance segmentation, providing a complete understanding of both, object instances and background categories.

In the context of deep learning, segmentation is typically achieved using Convolutional Neural Networks (CNN) or advanced architectures such as Mask R-CNN (He et al., 2020), which extends object detection models to output pixel-level masks representing the contour and interior for each detected object. This capability is especially valuable in agricultural and livestock settings, where animals may step on each other, move unpredictably, or appear in cluttered backgrounds.

In swine farming, instance segmentation can be used to identify and track individual pigs and their interactions over time (Zhang et al., 2019; Huang et al., 2023; Tu et al., 2024; Reza et al., 2025). When combined with deep learning-based classification, this approach enables precise detection of behaviours such as mounting. By extracting features such as posture, body orientation, and proximity between animals, the system can distinguish mounting from other social or exploratory behaviours. This not only automates labor-intensive monitoring tasks but also supports early intervention strategies to improve animal welfare and management practices.

A key advantage of deep learning is its ability to automatically detect and extract relevant features without explicitly defining search criteria typically needed in conventional machine learning approaches (Olden et al., 2008). Notably, deep learning has been shown to match or even exceed human performance in image recognition tasks, making it a promising tool for the automated detection of animal behaviours (He et al., 2016; Wu et al., 2016).

An essential aspect of machine and deep learning modeling is how data is structured and aligned with the model’s intended use. Before developing models, video information must be divided into training, validation, and testing datasets based on clips, frames, animals, days or locations. For instance, organizing data by frame, treats each frame as an independent data point, allowing frames from the same video to be placed in different datasets, while organizing by clip ensures that all frames from a given clip remain within the same dataset. Careful consideration is required in this process, as improper organization can lead to overly optimistic predictions, often caused by similar data, such as frames from the same clip being distributed across training and testing or between training and validations datasets. Additionally, when comparing results across studies, the data organization strategy must be considered, as performance measures are only comparable when the level of data granularity and separation is consistent.

Earlier work using computer vision and deep learning to detect mounting in pigs has typically relied on bounding-box detection (Faster R-CNN) with manually engineered geometric features (Ren et al., 2017; Yang et al., 2021; Li et al., 2019). The first study to apply an instance segmentation algorithm called Mask R-CNN to detect swine behaviour was conducted by Li et al. (2019) (Li et al., 2019). Mask R-CNN builds upon Faster R-CNN by generating an additional binary segmentation mask for each object, allowing for pixel-level segmentation and enhancing the model’s accuracy and flexibility for object detection. This study captured mounting behaviour in four miniature pigs, each weighing approximately 25 kg. They converted overhead video clips into individual frames, split the frames into training, validation, and testing, and generated eigenvectors representing half-body area, perimeter, and inter-object distance for each frame. These features were then used in a kernel extreme learning machine (KELM) model for behaviour classification. This approach achieved a sensitivity of 95.2%, a specificity of 88.3%, and an accuracy of 91.5% in detecting mounting behaviour on a testing set of 300 frames.

Building on prior research, our study introduces a hybrid approach that combines instance segmentation, deep learning and binary classification via traditional machine learning approaches to automatically detect mounting behaviour in boars. By enhancing both accuracy and efficiency, this research aims to create a practical, reliable solution for monitoring group-housed pigs. Additionally, this study examines how different data grouping methods affect machine learning model performance and generalization. Specifically, training, validation, and testing datasets were organized using three data grouping scenarios: by frame, by clip, and by day. We evaluated how each scenario affected the model’s ability to accurately detect mounting behaviour events, offering insights into the importance of data partitioning in machine learning workflows.

## 2 Materials and methods

### 2.1 Data collection and protocols

All experimental procedures adhered to the guidelines outlined by the Canadian Council of Animal Care (Canadian Council on Animal Care, 2009) and were approved by the University of Guelph Animal Care Committee (AUP #4600). The pilot study was conducted at the Ontario Swine Research Centre from March to May 2024.

### 2.2 Animals and housing

Eight Landrace × Yorkshire × Duroc crossbred boars were randomly selected from a group of 24 pigs available at the research center. No pre-screening for behavioural traits (e.g., known aggression or mounting tendency) was performed. All eight boars were free of clinical signs of illness determined during routine herd-health checks at the time of enrolment. The boars were between 4 and 5 months of age, each weighing between 60 and 90 kg and were uniformly white with no visible markings. The boars were randomly divided into two groups of four and housed in adjacent pens in a 23 °C room. For both pens, the dimensions were 1.53 m by 0.84 m, with 2.2-cm-thick and 51-cm-tall matte white plastic walls and fully slatted concrete floors. The distance from the camera to the floor was 1.31 m. The pens were illuminated with incandescent lights, and each contained a 0.89 m wide three-hole custom-made feeder and a bowl drinker. A see-saw enrichment toy with an attached chain was placed inside each pen.

In line with previous studies, overhead footage was used to capture a clear top-down view of the pigs, which minimizes occlusion and provides consistent visibility compared to side perspectives. The experimental setup closely mirrors commercial farming practices. According to the Canadian Code of Practice for the Care and Handling of Pigs (National Farm Animal Care Coun cil and Canadian Pork Council, 2014), boars are typically housed in individual pens. When grouped, they are placed in small, compatible cohorts, generally limited to three or four animals per pen. The minimum space allowance requires only enough room for the animals to lie down and turn around, and most commercial operations adhere closely to these requirements due to space constraints. Therefore, the conditions depicted in our videos are representative of real-world group-housed boar environments found on commercial farms.

### 2.3 Equipment and Data Acquisition

**FIGURE 1:**
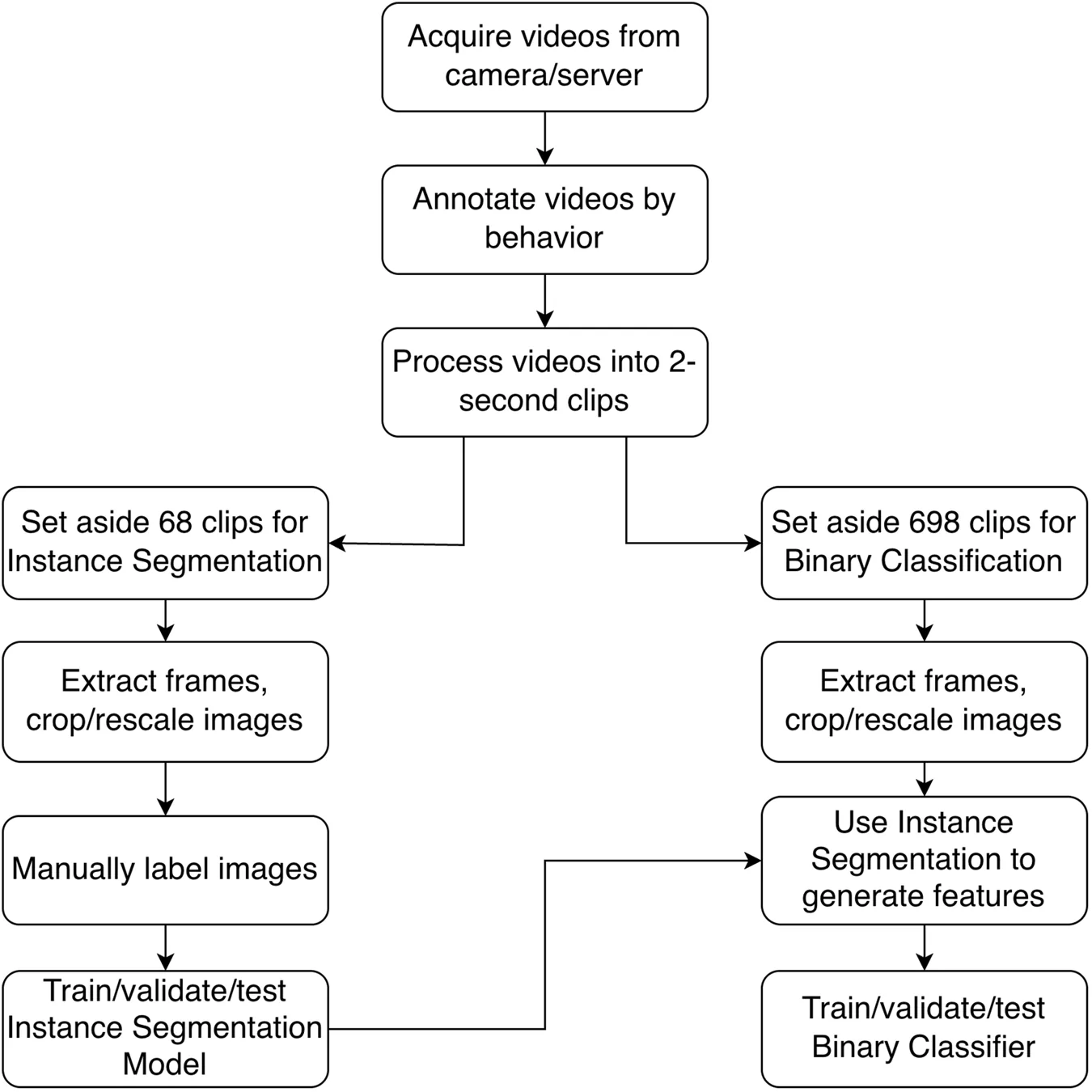
Workflow for mounting behaviour identification using machine and deep learning. The pipeline encompasses data acquisition, preprocessing, and parallel development of instance segmentation and binary classification models. Key steps include video annotation, clip extraction, frame processing, and dual model training/validation/testing, culminating in a hybrid system.

The trial videos were recorded using a top-down overhead view of both pens with a single Reolink TrackMix PoE surveillance camera (Shenzhen Reolink Technology Co., Ltd., New Castle, Delaware, United States). The camera was positioned above the middle divider between the two pens, 1.40 m from the back wall. It recorded continuously for 24 h a day for 1 week at a resolution of 3840 × 2160 pixels in full color at 15–25 frames per second (FPS), segmented into 30-min intervals. The videos were transferred to a local computer using a portable SSD drive. The computing system used for data processing and model development included an Intel(R) Core(TM) i7-14700F @ 2.10 GHz with 32 GB of RAM running Microsoft Windows 11 Enterprise with an NVIDIA GeForce RTX 4070 Ti SUPER. Programming the model pipeline components was performed in Python 3.9, TensorFlow Object Detection API 2.5, OpenCV 4.8.0.74, Scikit-learn 1.1.1, and Matplotlib 3.5.2. Figure 1 shows the end-to-end workflow for the mounting behaviour identification system from acquiring the video footage to binary classification.

### 2.4 Data preprocessing

Fifty video segments recorded over four separate days were annotated by an expert observer. The videos consisted of footage recorded during daylight hours when the pigs were visible, as no natural or artificial light was available at night. The observer reviewed each video segment using the VLC media player (VideoLAN, Paris, France) and manually recorded the start and end times of mounting and non-mounting behaviour to the nearest second in a spreadsheet. Mounting behaviour events lasting at least 2 s were recorded, based on findings from Chen et al. (2020) (Chen et al., 2020), which established this as the minimum time required for reliable behavioural identification. Mounting behaviour was defined as one pig using its legs to straddle the front or rear of another pig (Hintze et al., 2013).

The annotated videos were automatically segmented into standardized 2-s clips. From these, 766 clips were randomly selected and evenly split between mounting (383) and non-mounting (383) behaviours, to prevent the model from overestimating a majority class due to overrepresentation in the dataset. For instance segmentation training, 68 clips (∼9%) were set aside, with 34 clips reserved for training and 34 fully annotated validation frames (one frame from each of 34 separate clips) containing 272 ground-truth pig instances. Mounting and non-mounting clips were equally represented in both subsets. The remaining 698 clips were used for binary classification, including 349 (50%) with mounting behaviour and 349 (50%) with non-mounting behaviour.

A Group K-fold cross-validation strategy (3-fold) was applied to the training set, using three data grouping protocols: by frame, by clip, and by day. In the “by frame” protocol, each frame was treated as an independent sample, allowing frames from the same clip to appear in different dataset splits (training, validation or testing). In the “by clip” setup, entire clips comprising 30–50 frames were assigned exclusively to one dataset split, ensuring no overlap of frames from the same clip across training, validation, or testing. Similarly, the “by day” protocol restricted all data collected from a particular day to a single dataset split, so no two clips or frames from the same day were assigned to separate splits or folds. For example, all data collected from days 1, 2, and three were assigned to the training set, while all data from day 4 was placed in the testing set. During 3-fold cross-validation, 1 day was reserved for the validation fold while the other 2 days were assigned to the training folds. This process was repeated three times. For the first two scenarios (by frame and by clip), training contained 80% of the dataset while testing contained the remaining 20%. For the third scenario, 3 days were reserved for training and one for testing.

Each video clip was divided into individual frames and cropped from the original 3840 × 2160 pixels to 2800 × 2160 pixels to focus solely on the target pens and exclude irrelevant areas such as non-target pens or walkways. The images were then rescaled to 1024 × 1024 pixels. The model always processed a single frame at a time. The training and testing of the instance segmentation model were performed using one randomly selected frame per clip (from 68 clips representing 4 days of recordings) to ensure data diversity and improve computational efficiency. The frames were individually labeled using the Labelme software (Wada, 2018) by the same expert that performed behavioural annotation of the videos. Each pig was labeled with hand-drawn contours to generate segmentation masks. Pigs that were partially occluded were labeled based on their inferred position from visible parts and context from previous frames. Frames showing pigs distorted due to being at the edge of the lens’s field-of-view were included to help the model learn to recognize them. The annotations were saved as JSON files, paired with their corresponding images, and combined into *.record* files for model training, along with a model configuration (*.config)* file.

### 2.5 Instance segmentation

**FIGURE 2:**
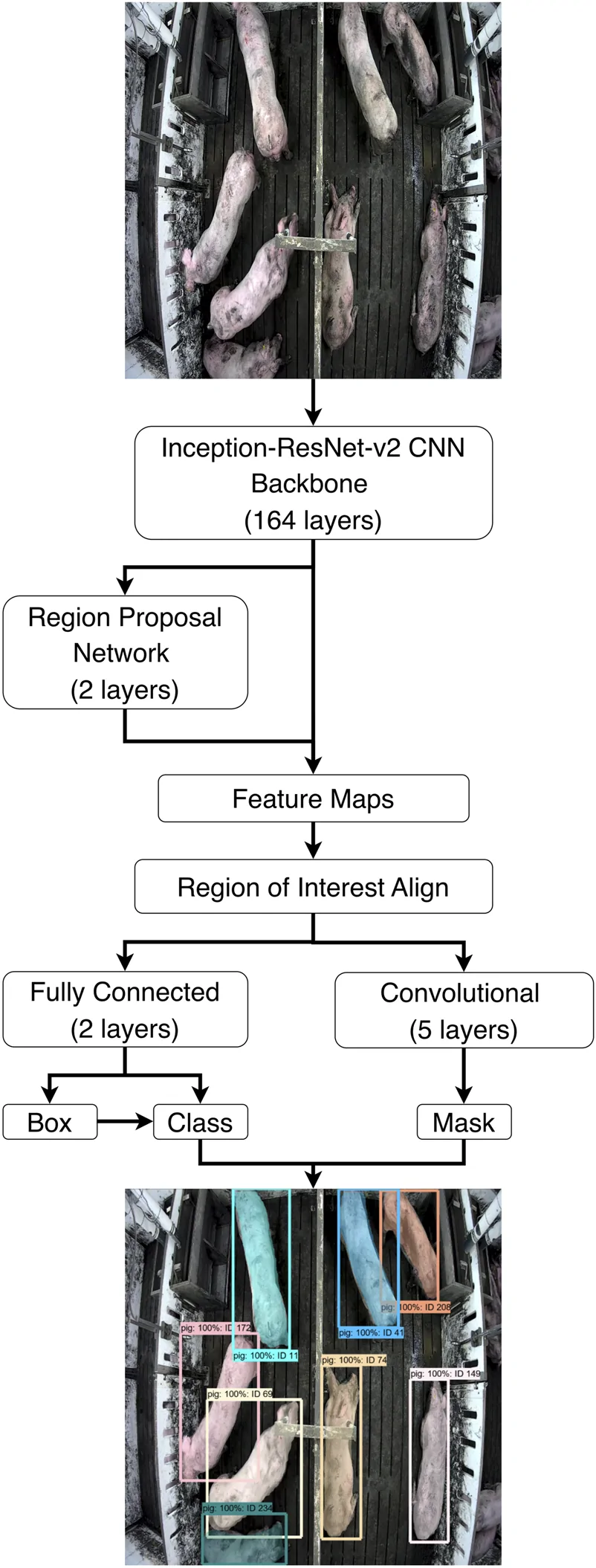
Schematic overview of the Mask R-CNN model for instance segmentation. The diagram illustrates the end-to-end pipeline, beginning with an input image processed by an Inception-ResNet-v2 CNN backbone to extract feature maps. These features are passed through a Region Proposal Network, followed by the Region of Interest Align layer, which ensures precise spatial alignment. The network then splits into two parallel output branches: a set of fully connected layers that generate object class labels and bounding boxes, and a convolutional branch that produces pixel-level segmentation masks. This extended framework enables simultaneous object detection and detailed pixel-level instance segmentation.

In this pilot study we implemented instance segmentation using the Mask R-CNN architecture (Figure 2) with an Inception-ResNet-v2 backbone consisting of 164 hidden layers (Szegedy et al., 2016) initialized with pre-trained ImageNet weights (Russakovsky et al., 2015). Mask R-CNN combines Region Proposal Networks (RPN) and Fully Convolutional Networks (FCN), allowing the generation of high-quality segmentation masks for each detected object (He et al., 2020). The Inception-ResNet-v2 model, used as the backbone for Mask R-CNN, integrates Inception modules with residual connections (Szegedy et al., 2016). Inception modules capture multi-scale features by processing the input frame at multiple scales in parallel, while residual connections help mitigate the vanishing gradient problem (Rehmer and Kroll, 2020) by allowing gradients to flow more effectively during training (Szegedy et al., 2016). Mask R-CNN is highly effective for object detection, generating bounding boxes around regions of interest surrounding detected objects, and performing instance segmentation by producing pixel-level masks that capture both the interior and contours of detected objects. These features were used to identify mounting behaviour, and for simplicity, the Mask R-CNN algorithm will be referred to as instance segmentation throughout this study.

The Mask R-CNN architecture processes input images through a multi-stage pipeline. First, the backbone Convolutional Neural Network extracts high-level feature maps from the input image representing abstract and semantic information about the image, learned from lower-level features. These feature maps are then passed to the Region Proposal Network, which identifies candidate object regions defined by bounding boxes by distinguishing them from the background. Each proposed region is then resized and processed by the Region of Interest Align component, which accurately extracts features by avoiding quantization errors during region resizing, ensuring precise spatial alignment for mask predictions. The aligned regions are subsequently passed through the Fully Connected Network, which predicts segmentation masks at the pixel level for each proposed region. The final output includes bounding boxes, class labels, prediction confidence scores, and binary masks that delineate the content and shape of each detected object in the image.

Instance segmentation was developed using the TensorFlow Object Detection API, a robust and flexible framework designed for training and deploying object detection and segmentation models. The architecture selected was the Inception-ResNet-v2 Mask R-CNN, which combines the feature extraction capabilities of Inception modules with the deep residual connections (Szegedy et al., 2016), providing a strong backbone for complex visual recognition tasks.

Key adjustments to the Inception-Resnet-v2 Mask-R-CNN were made to tailor the model to the specific task of detecting pigs in mounting and non-mounting scenarios. First, the number of object classes was set to one, as the objective was to first segment pigs regardless of behaviour type. The batch size was set to one due to GPU memory constraints and to allow for more precise updates with each image during training. Apart from these adjustments, the remaining model parameters, including anchor scales, learning rate, and optimizer settings, used their default settings provided by the TensorFlow API. The model was trained for 100 epochs, which was determined to be sufficient for convergence based on training and validation loss patterns. This setup enabled the model to learn high-quality instance segmentation masks, essential for accurately identifying individual pigs in complex group housing environments.

The instance segmentation dataset was used for transfer learning, a process in which a pre-trained model is adapted to a new, domain-specific task. In this study, the Mask R-CNN model pre-trained on ImageNet and COCO datasets, was fine-tuned by retraining its final layers using the custom-labeled pig behaviour dataset. This allowed the model to retain general visual features learned from large-scale datasets while becoming specialized in detecting pigs in group-housed environments.

After training, the customized instance segmentation model was applied to the full dataset to detect pigs across all video frames. Inference was performed on each individual frame within the video clips, yielding object detection results that included bounding boxes, segmentation masks, class labels, and prediction scores. The prediction-score threshold for retaining detections was set at the maximum SoftMax output (1.0). This deliberately strict criterion was chosen for two reasons specific to the downstream feature engineering. First, it suppresses double-detections of the same pig, which would otherwise inflate the Intersection-over-Union feature without indicating mounting, and ensures that high IoU values reflect genuine inter-pig overlap. Second, when two mounting pigs saturate to a single merged detection, the resulting drop in mask count and total mask-pixel area provides a complementary mounting signal that the binary classifier exploits. Because the classifier operates on the combined feature vector (mask count, total mask pixels, IoU, and inter-pig distances), occasional missed detections at the segmentation stage are recovered downstream rather than being lost as labels. We also evaluated the segmentation model independently, with no score floor, using these standard instance-segmentation measures (Results, Table 1), so the detector’s precision and recall behaviour across IoU thresholds can be inspected separately from the threshold used in the hybrid pipeline.

**TABLE 1:** Instance-segmentation performance of the Mask R-CNN pig detector, reported overall (the row labelled All true-positive instances) and stratified by occlusion level.

Group	n_GT	n_TP	Recall	Mask IoU	Dice/F1	mAP	AP@.50	AP@.75	AR
All true-positive instances	272	258	94.9 ± 1.1	81.8 ± 8.2	89.7 ± 5.4	61.0 ± 1.0	91.9 ± 1.1	75.2 ± 1.7	65.4 ± 1.0
Occlusion: none (<1%)	140	140	100.0 ± 0.0	85.4 ± 3.7	92.1 ± 2.2	71.3 ± 0.8	99.9 ± 0.1	96.9 ± 1.6	75.6 ± 0.6
Occlusion: low (1%–25%)	63	61	96.8 ± 2.2	82.8 ± 7.0	90.4 ± 4.5	61.4 ± 3.1	92.1 ± 3.0	77.3 ± 6.4	68.6 ± 2.5
Occlusion: moderate (25%–50%)	40	37	92.5 ± 5.2	72.4 ± 10.0	83.6 ± 6.9	35.3 ± 5.4	70.8 ± 7.1	25.8 ± 8.8	49.0 ± 4.6
Occlusion: heavy (>50%)	29	20	69.0 ± 9.2	70.6 ± 8.0	82.5 ± 5.7	20.7 ± 5.2	44.4 ± 10.2	13.4 ± 5.5	32.1 ± 5.0

The model was evaluated on a validation set of 34 fully annotated frames, one frame taken from each of 34 separate video clips, containing 272 ground-truth pig instances in total. Detections were matched to ground-truth pigs using standard mask-based matching: detections were considered in descending order of confidence with no confidence floor, and a detection counted as a true positive when its mask intersection over union (IoU) with a ground-truth pig was at least 0.5. Occlusion level is defined for each ground-truth pig as the fraction of its mask area overlapped by neighbouring pigs, and is binned as none (less than 1%), low (1%–25%), moderate (25%–50%) and heavy (greater than 50%). The reported measures are: n_GT, the number of ground-truth pigs in the group; n_TP, the number of those detected as true positives; Recall, equal to n_TP, divided by n_GT; Mask IoU, the mean IoU between predicted and ground-truth masks over the true-positive instances; Dice/F1, the mean Dice coefficient (the pixel-level F1 score) over the true-positive instances; mAP, the mean Average Precision averaged over ten IoU thresholds from 0.50 to 0.95; AP@.50 and AP@.75, the Average Precision at IoU thresholds of 0.50 and 0.75; and AR, the Average Recall allowing up to 100 detections per image. Recall, mAP, AP@.50, AP@.75 and AR, are computed over all ground-truth pigs (n_GT), whereas Mask IoU and Dice/F1 are averaged only over the detected true-positive instances (n_TP). All values are percentages, reported as mean ± standard deviation; the standard deviations for Mask IoU and Dice/F1 are computed across true-positive instances, while those for Recall, mAP, AP@.50, AP@.75 and AR, are image-level bootstrap estimates obtained from 1000 resamples of the 34 frames.

**FIGURE 3:**
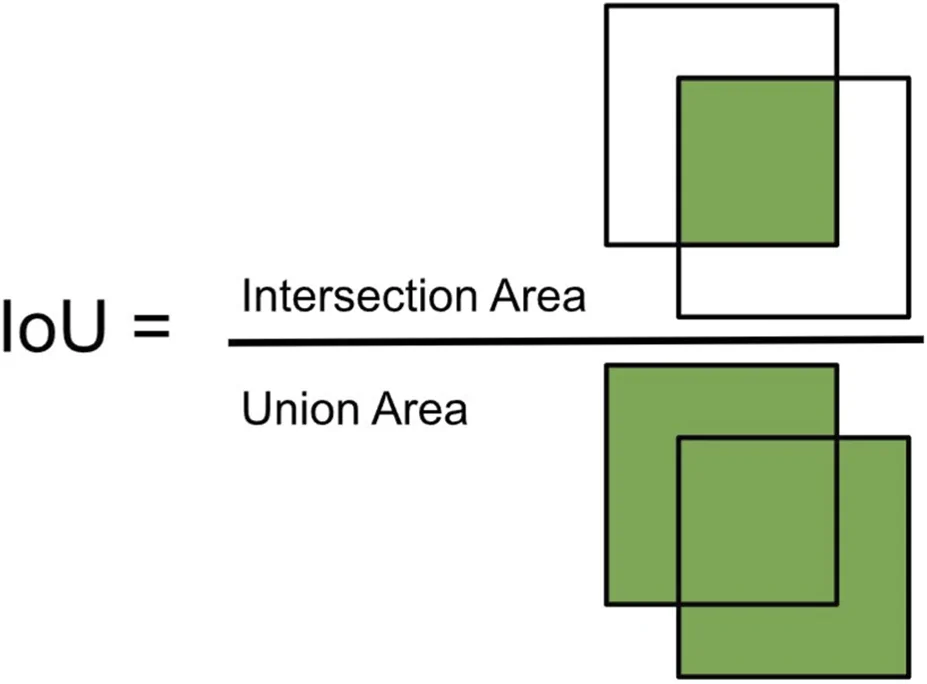
Graphical representation of Intersection over Union (IoU). IoU is a widely used measure that quantifies the degree of overlap between two regions, calculated as the ratio of their intersection area to their combined union area. Its values range from 0 (no overlap) to 1 (complete overlap). In the context of this study, IoU is derived from the instance segmentation outputs by calculating the overlap between the predicted masks of detected pigs. This measure was used both as a predictive feature for binary classification, representing the extent of vertical occlusion in frames, and as a performance evaluation measure to assess the accuracy of the instance segmentation model by comparing predicted masks with expert-labeled ground-truth.

The detection results included bounding boxes, masks, labels, and prediction scores for each pig. To extract meaningful features for downstream behaviour classification, several spatial and morphological measures were calculated. The Intersection over Union (IoU) was calculated across all detected masks in each frame to quantify the proportion of mask overlap, which served as an indicator of vertical occlusion as a feature illustrated in Figure 3. Additionally, Euclidean distances were calculated between the centroids of all bounding boxes. For example, when eight pigs were detected, this produced 28 unique pairwise distance values, capturing spatial proximity and clustering patterns among pigs.

For each processed image, a total of nine features were extracted and compiled into a structured tabular format suitable for binary classification. These features included: the total number of masks representing detected pigs, the total number of pixels covered by all masks delineating the aggregate pig body area, the IoU values of overlapping masks, the minimum, maximum, and average pairwise distances between pigs, and first (Q1), second (Q2) and third (Q3) quartiles of the distribution of distances among pigs.

### 2.6 Instance segmentation evaluation

To rigorously assess the performance of the trained instance segmentation models, four primary evaluation measures were utilized: Intersection over Union (IoU), mean Average Precision (mAP), Average Recall (AR), and the Dice Coefficient (DICE score). These measures collectively evaluate the models' capabilities in both object detection (localization) and pixel-level classification (segmentation).

**Intersection over Union (IoU)**: Intersection over Union (IoU), also known as the Jaccard Index, quantifies the overlap between the predicted segmentation mask ( A ) and the ground truth mask ( B ). It is calculated as the ratio of the area of intersection to the area of union between the two masks:

$$IoU=\frac{\left|A\cap B\right|}{\left|A\cup B\right|}$$

An IoU score of 1.0 indicates perfect overlap, while 0.0 indicates no overlap. In instance segmentation, a prediction is typically classified as a True Positive (TP) if its IoU exceeds a predefined threshold (e.g., IoU≥0.5 ).

**Mean Average Precision (mAP)**: Precision measures the proportion of true positive detections among all positive predictions made by the model. Average Precision (AP) computes the area under the precision-recall curve for a single class. For multi-class instance segmentation, performance is aggregated using the mean Average Precision (mAP), which averages the AP across all N distinct classes:

$$mAP=\frac{1}{N}\sum_{i=1}^{N}{AP}_{i}$$

where ${AP}_{i}$ represents the AP of each class i .

In accordance with standard benchmarks (such as the COCO dataset), this study reports mAP@[0.50:0.95], which represents the average mAP calculated across ten evenly spaced IoU thresholds from 0.50 to 0.95 with a step size of 0.05. This penalizes poor spatial alignment and rewards highly accurate mask boundaries.

**Average Recall (AR)**: Recall measures the model’s ability to identify all relevant ground truth instances within the dataset. Average Recall (AR) is computed as the maximum recall given a specific number of detections per image, averaged over the standard suite of IoU thresholds ranging from 0.50 to 0.95:

$$AR=2\int_{0.50}^{0.95}Recall\left(t\right)dt$$

where t represents the IoU threshold. This measure evaluates the model’s sensitivity and its robustness against missing target objects.

**Dice Coefficient (DICE Score)**: The Dice Coefficient, or F1-score at the pixel level, measures the harmonic mean of precision and recall. It is closely related to IoU but places a higher mathematical weight on the intersection of the predicted ( A ) and ground truth (B) masks:

$$DICE=\frac{2\left|A\cap B\right|}{\left|A\right|+\left|B\right|}$$

The DICE score is particularly sensitive to severe class imbalances (e.g., small foreground instances against a large background) and ranges from 0 (no overlap) to 1 (perfect segmentation alignment).

These measures were computed on the validation set of 34 fully annotated frames (272 ground-truth pig instances) described above. For each image, predicted instances were considered in descending order of confidence and matched to ground-truth pigs, with no confidence threshold applied; a prediction was counted as a true positive when its mask IoU with an unmatched ground-truth pig was at least 0.5. Under this matching, 258 of the 272 ground-truth instances were matched, leaving 14 missed detections. Recall, mAP, AP@.50, AP@.75 and AR were computed over all ground-truth instances, whereas mean mask IoU and Dice/F1 were averaged only over the matched true-positive instances, because mask overlap is undefined for an undetected pig.

To characterize robustness to crowding, each ground-truth pig was assigned an occlusion level computed directly from the annotation polygons, which were rasterized to binary masks at full image resolution, as the fraction of its mask area overlapped by the union of the other pig masks in the same frame. Instances were grouped into four levels, none (less than 1%), low (1%–25%), moderate (25%–50%) and heavy (greater than 50%), and every measure was recomputed within each group.

Because the two families of measures differ statistically, their standard deviations were obtained in two ways. Mean mask IoU and Dice/F1 are per-instance quantities, so the reported standard deviation is the ordinary spread across the true-positive instances in each group. Recall, mAP, AP@.50, AP@.75 and AR are aggregate measures that yield a single value per evaluation, so their standard deviations were estimated by an image-level bootstrap, in which the 34 frames were resampled with replacement and each measure recomputed over 1000 resamples, with the standard deviation taken across those resamples.

### 2.7 Binary classification

The nine features derived from the output of the instance segmentation model served as inputs for the subsequent binary classification. Prior to model training, these features were standardized using z-score normalization to achieve a mean of zero and a standard deviation of one. This process eliminated differences in scale across features while preserving the shape of their distribution.

Five binary ML classification models were selected based on their popularity and suitability for structured tabular data: Gradient Boosting (GB), Random Forest (RF), Logistic Regression (LR), Multi-Layer Perceptron (MLP), and Support Vector Machine (SVM). GB is an ensemble method that builds multiple decision trees sequentially, where each new tree corrects the residual errors of its predecessors, effectively minimizing a loss function through gradient descent (Friedman, 2001). RF is another ensemble technique that constructs multiple decision trees during training, then aggregates their outputs by majority vote for classification, enhancing robustness and reducing overfitting (Breiman, 2001). LR estimates the probability of a binary outcome using a logistic function and is especially effective for linearly separable problems (Zhu et al., 1997). Support Vector Machine (SVM) determines the optimal hyperplane that best separates classes in the feature space, maximizing the margin between them. For data that is not linearly separable, SVM applies a kernel function to transform the feature space into a higher-dimensional space where separation becomes feasible (Cortes and Vapnik, 1995). MLP is a type of feedforward artificial neural network with multiple layers of interconnected neurons. It captures complex non-linear relationships in the data through multiple hidden layers and activation functions, enabling the model to learn intricate patterns (Rosenblatt, 1958).

### 2.8 Binary model evaluation

The binary ML classifiers used in this study were evaluated using a comprehensive set of performance measures designed to assess both overall performance and class-specific prediction quality. These measures include balanced accuracy, precision, recall (also known as TPR = True Positive Rate), and F1-score, which were calculated using the following equations:

$$Precision=\frac{TP}{TP+FP}$$

$$Recall\;or\;TPR=\frac{TP}{TP+FN}$$

$$TNR=\frac{TN}{\left(TN+FP\right)}$$

$$Balanced\;Accuracy=\frac{TPR+TNR}{2}$$

$$F1\;Score=2\times \frac{Precision\times Recall}{Precision+Recall}$$

where true positives (TP) refer to mounting episodes correctly identified as mounting, true negatives (TN) denote non-mounting episodes correctly identified as non-mounting, false positives (FP) indicate non-mounting episodes mistakenly classified as mounting, and false negatives (FN) are mounting episodes incorrectly classified as non-mounting.

Balanced accuracy is especially important in cases where classes are imbalanced or of unequal importance, as it accounts for both sensitivity or True Positive Rate (TPR) and specificity or True Negative Rate (TNR), ensuring a fairer evaluation across classes.

To monitor the learning process and detect overfitting during model training, the binary cross-entropy (BCE) loss function was used:

$$BCE=-\frac{1}{N}\;\sum_{i=1}^{N}y_{i}\cdot \log\left(p\left(y_{i}\right)\right)+\left(1-y_{i}\right)\cdot \log\left(1-p\left(y_{i}\right)\right)$$

where $y_{i}$ represents the class label, log(*p*(*y_i_*)) represents the natural logarithm of the predicted probability of that class, $p\left(y_{i}\right)$ is the probability of mounting, and $1-p\left(y_{i}\right)$ is the probability of non-mounting.

BCE assigns a greater penalty to incorrect predictions, resulting in higher values for misclassifications and lower values for correct classifications. Therefore, a lower BCE loss corresponds to better model performance, while higher values may indicate misclassification or overfitting.

The performance of the instance segmentation model, which was responsible for identifying and isolating individual pigs in each frame, was assessed by measuring the preciseness of object localization through calculating the IoU between the ground truth masks and the predicted masks.

To optimize model performance, hyperparameter tuning was conducted using a grid search strategy and 3-fold cross-validation (Pedregosa et al., 2015), based on balanced accuracy. This process systematically explored combinations of key hyperparameters (e.g., learning rate, regularization strength, kernel type) to identify the configuration that yielded the highest balanced accuracy on the validation data.

Training the instance-segmentation model required 100 epochs and a total of 285 min (4 h 45 min) on the system described in Equipment and Data Acquisition. Binary classification training times varied by algorithm, ranging from 15 s per replicate for Logistic Regression (quickest) to 65 s per replicate for Support Vector Classification (slowest).

### 2.9 Fitting analysis

Learning curves were employed to evaluate model fitting behaviour and generalization performance under varying data partitioning strategies. These curves plot balanced accuracy on the y-axis against the number of training samples on the x-axis for both training and validation datasets. By visualizing the progression of performance over time, learning curves serve as a diagnostic tool to assess model convergence, detect underfitting or overfitting, and guide decisions regarding model complexity, regularization, and data sufficiency. Learning curves were generated separately for each data grouping strategy − by frame, by clip, and by day − to characterize how differences in data granularity affect the learning dynamics of the classification model. This approach enables comparative assessment of training stability and generalization capacity across scenarios.

### 2.10 Model explainability

Model explainability was assessed with Shapley Additive Explanations (SHAP), which is a unified framework for explaining the output of machine learning models (Lundberg et al., 2017). SHAP assigns each feature a contribution score based on game theory, illustrating how much each feature contributes to a particular prediction. The resulting visualizations use color to represent the magnitude of feature values (red for high, blue for low), and the x-axis to indicate the direction and magnitude of the feature’s effect on the model’s output. Additionally, the higher a feature’s position on the y-axis is, the more important it is to the model’s predictive capabilities relative to other features. This technique provided insight into which features were most influential in detecting mounting behaviour.

## 3 Results and discussion

### 3.1 Instance segmentation performance

The Mask R-CNN instance-segmentation model was evaluated on a validation set of 34 fully annotated frames containing 272 ground-truth pig instances, using standard instance-segmentation measures (Table 1). Of the 272 pigs, 258 were correctly detected at a mask IoU of at least 0.5, an overall recall of 94.9%. On these true positives the mean mask IoU was 81.8% and the mean Dice/F1 was 89.7%, while detection quality over the whole set reached a mean Average Precision (mAP) of 61.0%, with AP of 91.9% at an IoU threshold of 0.50% and 75.2% at 0.75, and an Average Recall (AR) of 65.4%.

Performance degraded monotonically with occlusion across every measure (Table 1). Recall fell from 100% for unoccluded pigs to 69.0% under heavy occlusion (more than half of the mask overlapped by neighbouring pigs), and AP at IoU 0.50 dropped from 99.9% to 44.4% over the same range. Segmentation quality proved more robust than detection: even under heavy occlusion the mean Dice/F1 remained 82.5% and the mean mask IoU 70.6%, so the limiting factor for heavily occluded pigs is detection rather than mask precision, with 9 of the 14 missed pigs falling in the heavy-occlusion bin. This is the condition the downstream binary classifier is designed to exploit through complementary mask-count and pixel-area features.

**FIGURE 4:**
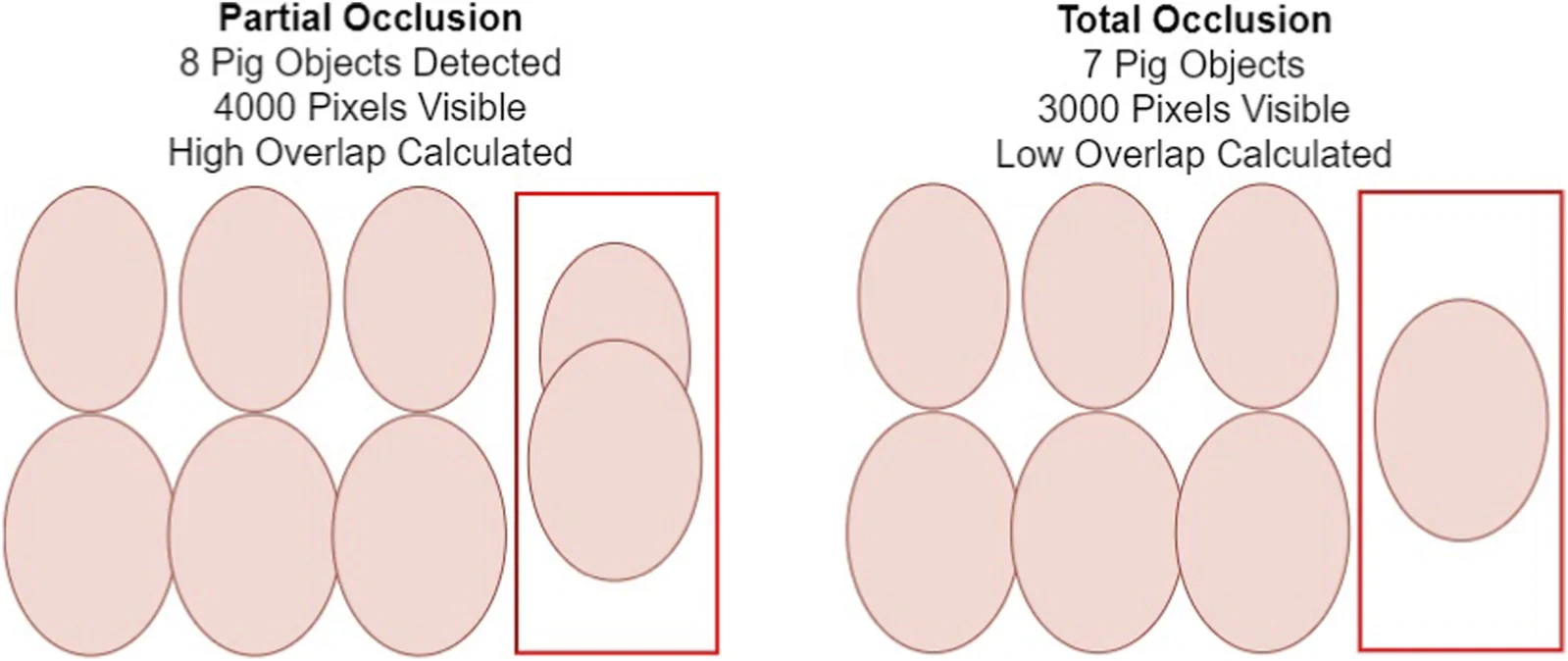
Graphical representation of partial versus total occlusion of objects. The scenario on the left depicts 8 pigs represented as pink ovals, with a simulated mounting event surrounding by a rectangle with a red outline. This mounting event shows a pig partially occluding another one (both pigs still being visible) with a total of 4,000 visible pixels. The scenario on the right shows a total occlusion scenario where the mounting event shows only one visible pig. This is due to the other pig being totally occluded underneath the mounting pig, resulting in less pigs and pixels.

In cases where segmentation errors occurred, such as merged, split, or incorrectly detected pigs, the model leveraged variations and interactions among features such as IoU, number of masks, number of pixels, and inter-pig distances to adjust and maintain robust predictions. For example, during a typical mounting event involving two pigs with partial occlusion, high overlap (high IoU), 8 detected objects and 4,000 mask pixels can be observed (Figure 4). In contrast, during total occlusion, when the mounting pig fully obscures the mounted pig, the number of detected objects drops to 7, with reduced overlap and a decrease in pixel count to around 3000 due to the mounted pig being undetected. Although a lack of overlap typically suggests non-mounting, the reduced mask pixel area in this case suggests mounting behaviour because it indicates a hidden pig. The interaction of these features allows the model to compensate for segmentation inaccuracies and enhances its overall behavioural classification performance.

### 3.2 Binary classification performance

SVM was selected as the best overall algorithm, achieving the highest average testing score for balanced accuracy (0.90) and F1 score (0.89) across all three scenarios (Table 2). RF followed closely with a balanced accuracy of 0.90, while GB and MLP scored slightly lower at 0.88 and 0.83, respectively. LR generally underperformed, particularly in high-variance or low-data scenarios with an average balanced accuracy of 0.78.

**TABLE 2:** Results of binary classification for five algorithms (Gradient Boosting [GB], Random Forest [RF], Logistic Regression [LR], Multi-Layer Perceptron [MLP], Support Vector Machine [SVM]) under three data-grouping scenarios (by frame, by clip, by day). Reported values are means across the 3-fold cross-validation. BCE = binary cross-entropy.

Grouped by	Measure	Dataset split	GB	RF	LR	MLP	SVM
Frame	Balanced Accuracy	Train	1.00 ± 0.00	1.00 ± 0.00	0.81 ± 0.00	1.00 ± 0.00	1.00 ± 0.00
		Val	1.00 ± 0.00	1.00 ± 0.00	0.81 ± 0.00	1.00 ± 0.00	0.99 ± 0.00
		Test	1.00 ± 0.00	1.00 ± 0.00	0.81 ± 0.00	1.00 ± 0.00	1.00 ± 0.00
	Precision	Train	1.00 ± 0.00	1.00 ± 0.00	0.83 ± 0.00	1.00 ± 0.00	1.00 ± 0.00
		Val	1.00 ± 0.00	1.00 ± 0.00	0.83 ± 0.00	1.00 ± 0.00	0.99 ± 0.00
		Test	1.00 ± 0.00	1.00 ± 0.00	0.83 ± 0.01	1.00 ± 0.00	1.00 ± 0.00
	Recall	Train	1.00 ± 0.00	1.00 ± 0.00	0.78 ± 0.00	1.00 ± 0.00	0.99 ± 0.00
		Val	1.00 ± 0.00	1.00 ± 0.00	0.78 ± 0.01	1.00 ± 0.00	0.99 ± 0.00
		Test	1.00 ± 0.00	1.00 ± 0.00	0.76 ± 0.01	1.00 ± 0.00	1.00 ± 0.00
	F1 score	Train	1.00 ± 0.00	1.00 ± 0.00	0.81 ± 0.00	1.00 ± 0.00	1.00 ± 0.00
		Val	1.00 ± 0.00	1.00 ± 0.00	0.81 ± 0.00	1.00 ± 0.00	0.99 ± 0.00
		Test	1.00 ± 0.00	1.00 ± 0.00	0.79 ± 0.00	1.00 ± 0.00	1.00 ± 0.00
	BCE	Train	0.00 ± 0.00	0.01 ± 0.00	0.40 ± 0.00	0.00 ± 0.00	0.02 ± 0.00
		Val	0.01 ± 0.00	0.02 ± 0.00	0.41 ± 0.01	0.01 ± 0.00	0.02 ± 0.00
		Test	0.01 ± 0.00	0.02 ± 0.00	0.41 ± 0.01	0.01 ± 0.00	0.01 ± 0.00
Clip	Balanced Accuracy	Train	1.00 ± 0.00	1.00 ± 0.00	0.82 ± 0.01	1.00 ± 0.00	1.00 ± 0.00
		Val	0.96 ± 0.00	0.96 ± 0.01	0.81 ± 0.01	0.96 ± 0.01	0.96 ± 0.01
		Test	0.96 ± 0.01	0.95 ± 0.01	0.78 ± 0.00	0.96 ± 0.01	0.95 ± 0.01
	Precision	Train	1.00 ± 0.00	1.00 ± 0.00	0.83 ± 0.02	1.00 ± 0.00	1.00 ± 0.00
		Val	0.95 ± 0.01	0.94 ± 0.02	0.83 ± 0.02	0.95 ± 0.02	0.94 ± 0.01
		Test	0.95 ± 0.03	0.95 ± 0.03	0.80 ± 0.01	0.96 ± 0.02	0.94 ± 0.03
	Recall	Train	1.00 ± 0.00	1.00 ± 0.00	0.78 ± 0.01	1.00 ± 0.00	1.00 ± 0.00
		Val	0.97 ± 0.01	0.97 ± 0.01	0.78 ± 0.02	0.97 ± 0.01	0.97 ± 0.01
		Test	0.97 ± 0.02	0.96 ± 0.02	0.74 ± 0.02	0.97 ± 0.03	0.96 ± 0.03
	F1 score	Train	1.00 ± 0.00	1.00 ± 0.00	0.81 ± 0.01	1.00 ± 0.00	1.00 ± 0.00
		Val	0.96 ± 0.00	0.96 ± 0.01	0.81 ± 0.02	0.96 ± 0.01	0.96 ± 0.01
		Test	0.96 ± 0.01	0.95 ± 0.01	0.77 ± 0.01	0.96 ± 0.01	0.95 ± 0.00
	BCE	Train	0.00 ± 0.00	0.00 ± 0.00	0.40 ± 0.03	0.00 ± 0.00	0.01 ± 0.00
		Val	0.15 ± 0.04	0.11 ± 0.02	0.43 ± 0.06	0.18 ± 0.10	0.27 ± 0.14
		Test	0.16 ± 0.04	0.10 ± 0.01	0.43 ± 0.02	0.13 ± 0.03	0.20 ± 0.04
Day	Balanced Accuracy	Train	0.93 ± 0.02	0.92 ± 0.01	0.87 ± 0.04	1.00 ± 0.00	0.88 ± 0.04
		Val	0.67 ± 0.15	0.68 ± 0.11	0.63 ± 0.12	0.52 ± 0.08	0.64 ± 0.11
		Test	0.67 ± 0.19	0.74 ± 0.09	0.75 ± 0.02	0.54 ± 0.22	0.74 ± 0.01
	Precision	Train	0.92 ± 0.02	0.93 ± 0.01	0.88 ± 0.03	1.00 ± 0.00	0.89 ± 0.02
		Val	0.68 ± 0.22	0.72 ± 0.16	0.67 ± 0.19	0.58 ± 0.16	0.68 ± 0.17
		Test	0.67 ± 0.30	0.79 ± 0.16	0.79 ± 0.08	0.64 ± 0.35	0.79 ± 0.07
	Recall	Train	0.94 ± 0.03	0.93 ± 0.02	0.86 ± 0.05	1.00 ± 0.00	0.87 ± 0.06
		Val	0.70 ± 0.27	0.68 ± 0.25	0.80 ± 0.18	0.61 ± 0.29	0.79 ± 0.17
		Test	0.60 ± 0.20	0.62 ± 0.08	0.67 ± 0.03	0.29 ± 0.20	0.66 ± 0.03
	F1 score	Train	0.93 ± 0.02	0.93 ± 0.01	0.87 ± 0.04	1.00 ± 0.00	0.88 ± 0.04
		Val	0.67 ± 0.20	0.67 ± 0.17	0.69 ± 0.07	0.54 ± 0.14	0.70 ± 0.06
		Test	0.63 ± 0.25	0.69 ± 0.10	0.72 ± 0.03	0.38 ± 0.24	0.72 ± 0.02
	BCE	Train	0.34 ± 0.02	0.28 ± 0.03	0.33 ± 0.06	0.00 ± 0.00	0.35 ± 0.07
		Val	0.61 ± 0.10	0.59 ± 0.08	1.08 ± 0.71	6.40 ± 4.21	1.46 ± 1.81
		Test	0.58 ± 0.13	0.58 ± 0.09	0.55 ± 0.05	3.86 ± 2.67	0.62 ± 0.08

In Scenario 1, individual video frames were randomly assigned to training, validation, and testing sets. Testing performance appeared nearly perfect, with most models achieving between 0.99 and 1.00 across all measures, except LR, which scored between 0.76 and 0.83. However, this high performance was misleading. As videos were recorded at 15–25 FPS, adjacent frames often appeared in both training and testing sets, artificially inflating the model’s apparent generalization capability. Although technically not data leakage, and with consistent validation measures, this setup led to pattern memorization rather than true behaviour recognition. The model learned frame-level features rather than generalizable behavioural cues, failing to address variability such as lighting changes or different environmental conditions.

In Scenario 2, data was organized into 2-s clips before being split into training, validation and testing datasets. These clips corresponded to the standardized segments created during dataset design to normalize various behaviour durations while enabling class-balanced splits and model evaluation. No two frames from the same 2-s clip were assigned to different dataset splits (training, validation and testing). As a result, testing measures dropped slightly, with all models achieving accuracy, precision, recall, and F1 scores between 0.94 and 0.97, except LR, which achieved scores between 0.74 and 0.80 for the same measures. Although clips were distinct, some originated from the same continuous behavioural sequence, allowing limited information redundancy across splits. This scenario better approximated real-world conditions and revealed the models’ true performance potential.

In the most realistic setup (Scenario 3), data were partitioned by day - one for testing, three for training, and one per fold for validation. This resulted in highly distinct and imbalanced datasets that better reflect real-world model deployment. Testing balanced accuracy varied widely, ranging from 0.29 to 0.79, with LR unexpectedly achieving the highest balanced accuracy at 0.75, likely due to its simplicity and regularization capabilities. SVM and RF also performed well, each reaching 0.79 precision, while SVM achieved a balanced accuracy of 0.74. This scenario highlighted the importance of generalization, as models were evaluated on entirely unseen behaviour contexts and lighting conditions.

### 3.3 Feature importance and fitting analysis

**FIGURE 5:**
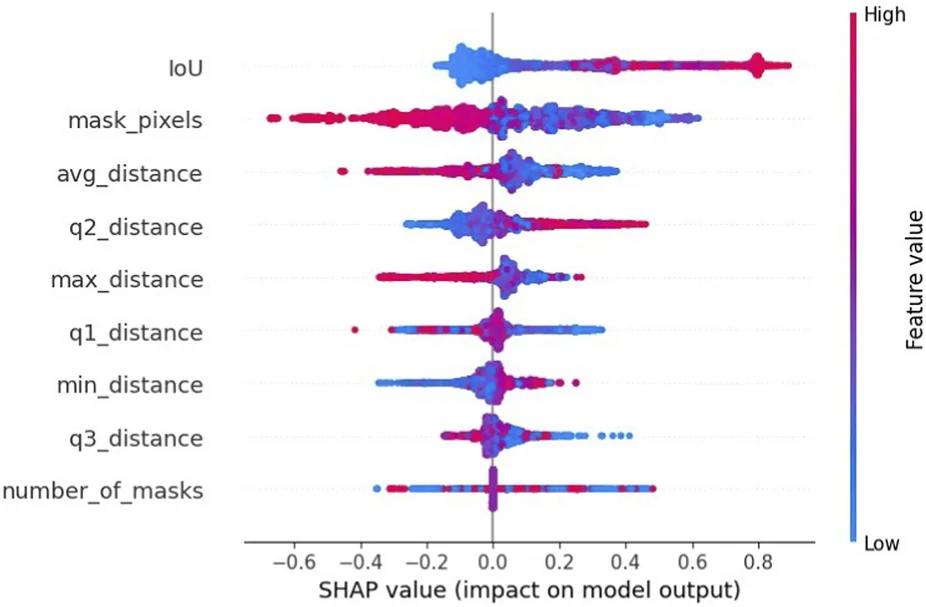
SHAP analysis of feature contributions to SVM model predictions for mounting behaviour. The SHAP summary plot illustrates the influence of input features on SVM model predictions, grouped by video clip. Feature values are color-coded: red indicates high, blue indicates low, and purple represents intermediate values. Movement to the right along the x-axis reflects a positive contribution to mounting predictions, while movement to the left indicates influence toward non-mounting predictions.

SHAP analysis (Figure 5) showed that, within this dataset, IoU was the most influential feature for the trained SVM, with high IoU values associated with mounting-class predictions and low IoU values associated with non-mounting-class predictions. Mask pixel count was the second most influential feature: low mask-pixel counts, typically arising from mask merging or partial occlusion, were associated with mounting class predictions, and high mask-pixel counts with non-mounting class predictions. These SHAP attributions describe associations learned by the model under the present experimental conditions rather than direct causal indicators of mounting behaviour itself.

**FIGURE 6:**
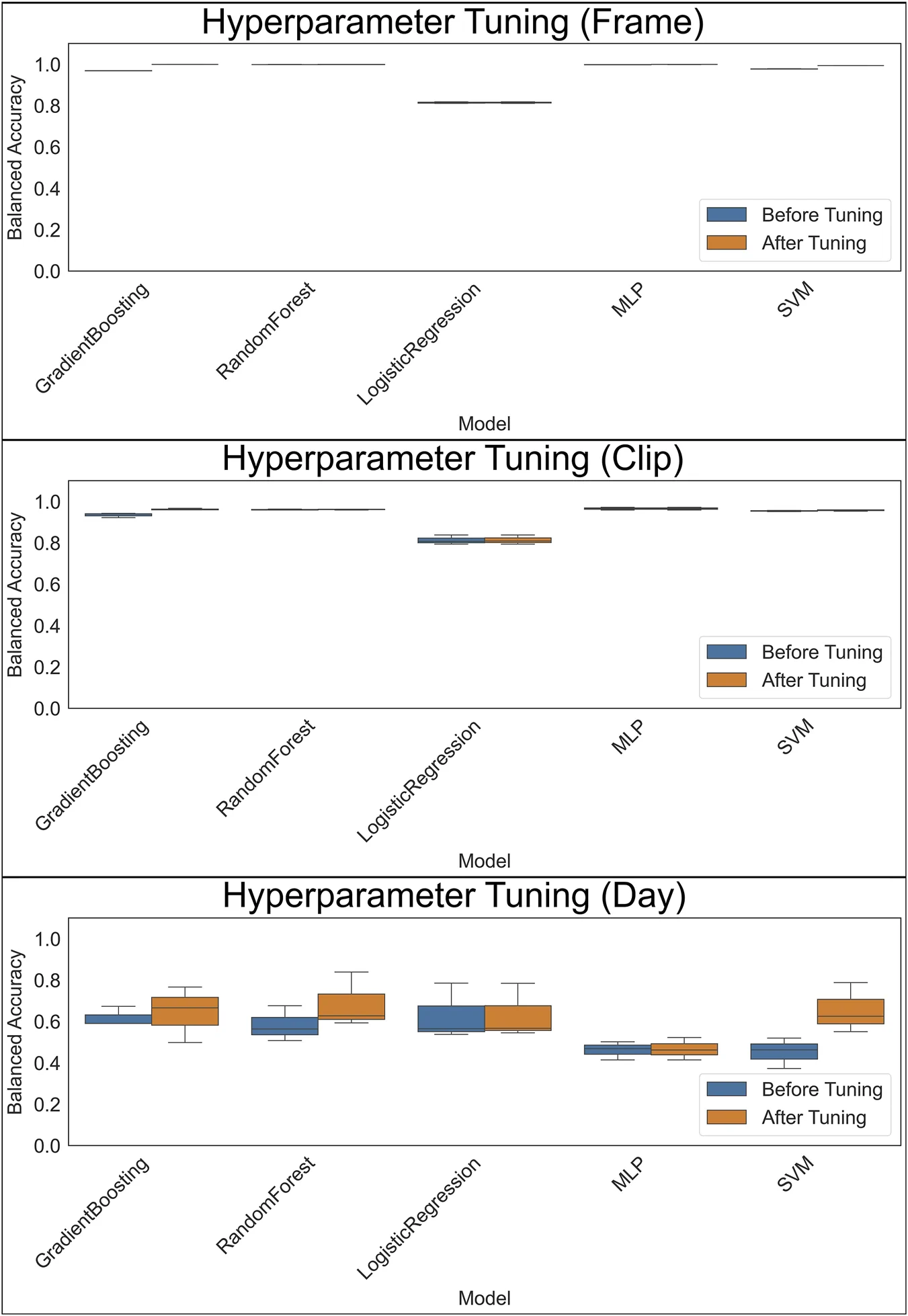
Comparative analysis of balanced accuracy for five machine learning models before and after hyperparameter tuning, across three data grouping scenarios. The box plots illustrate the distribution of balanced accuracy scores for 5 ML models - Gradient Boosting (GB), Random Forest (RF), Logistic Regression (LR), Multi-Layer Perceptron (MLP), and Support Vector Machine (SVM) - evaluated on a dataset with 698 video clips. Performance is compared before and after hyperparameter tuning under three distinct data grouping strategies: by individual frame, by 2-s clip, and by recording day. Each grouping scenario highlights how data partitioning influences model generalization and optimization outcomes.

Initial model evaluations used default hyperparameters, which were later refined using grid search with 3-fold cross-validation. Balanced accuracy guided the optimization performance. Scenario three benefited the most from tuning, with performance improvements indicating better adaptation to unseen data (Figure 6). Simpler, regularized models were preferred to reduce overfitting and maintain processing speed at or above 5 FPS.

**FIGURE 7:**
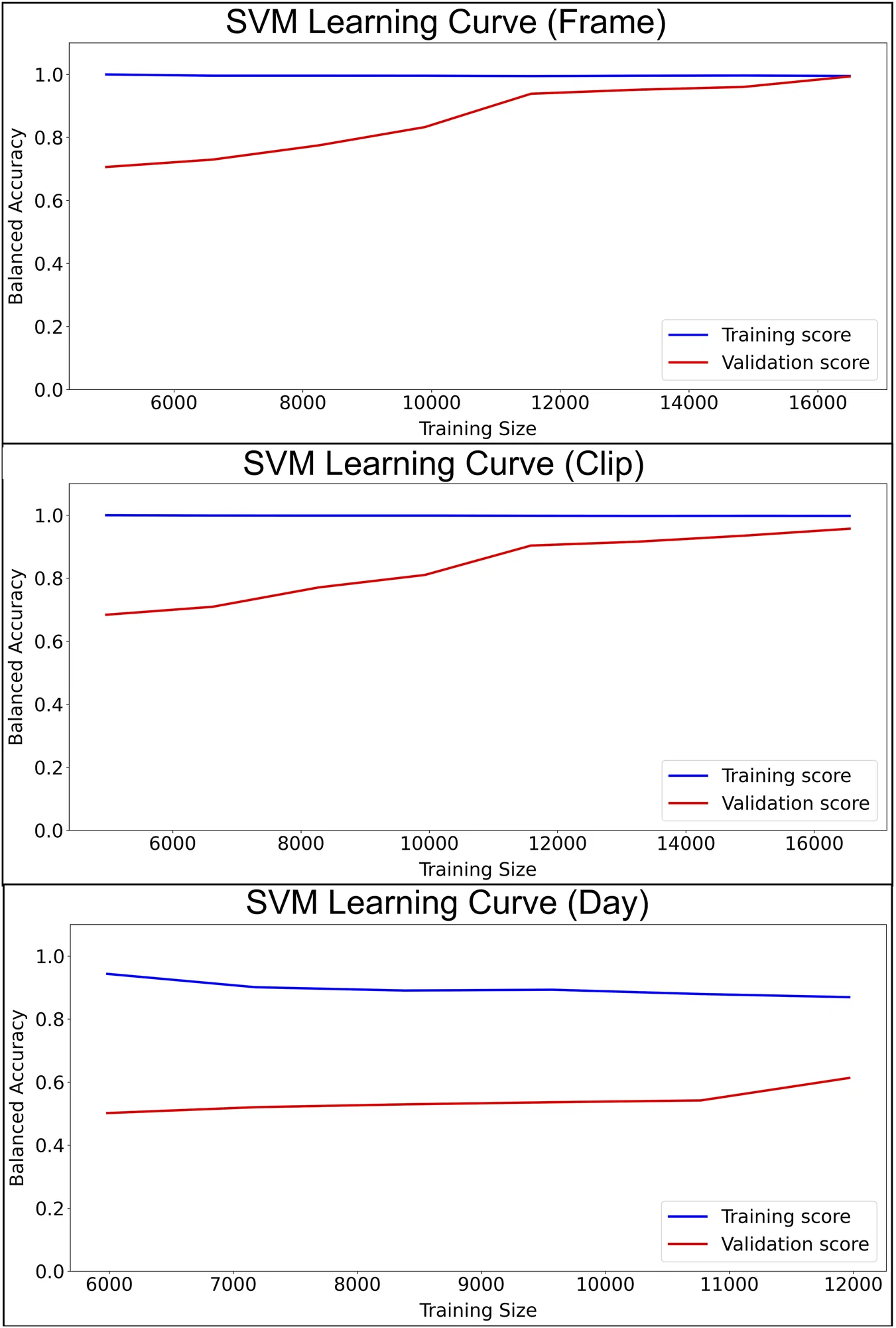
Comparison of SVM learning curves across three data grouping scenarios after hyperparameter optimization. The learning curves illustrate the training and 3-fold cross-validation performance of the Support Vector Machine (SVM) model, measured using balanced accuracy, across a training dataset of 698 video clips. The curves represent three distinct data grouping scenarios - by individual frame, by 2-s clip, and by recording day - demonstrating the impact of data partitioning on model learning dynamics, convergence behaviour, and generalization capability following hyperparameter tuning.

Although pigs and pens remained consistent across days, behavioural segments were unique across dataset splits. The limited availability of mounting events restricted testing to only 1 day, while the other 3 days were used for training and validation. However, as shown in the learning curve comparison (Figure 7), model training plateaued at 12,000 frames in all scenarios. This indicates that the available data was insufficient, as only four distinct days were recorded due to the infrequent occurrence of mounting behaviour, with half used for training and just 1 day used for validation and testing in each replicate. With additional training data spanning more days, the model could have progressed toward full convergence, increasing its ability to generalize by learning underlying behavioural patterns rather than relying on dataset-specific characteristics.

**FIGURE 8:**
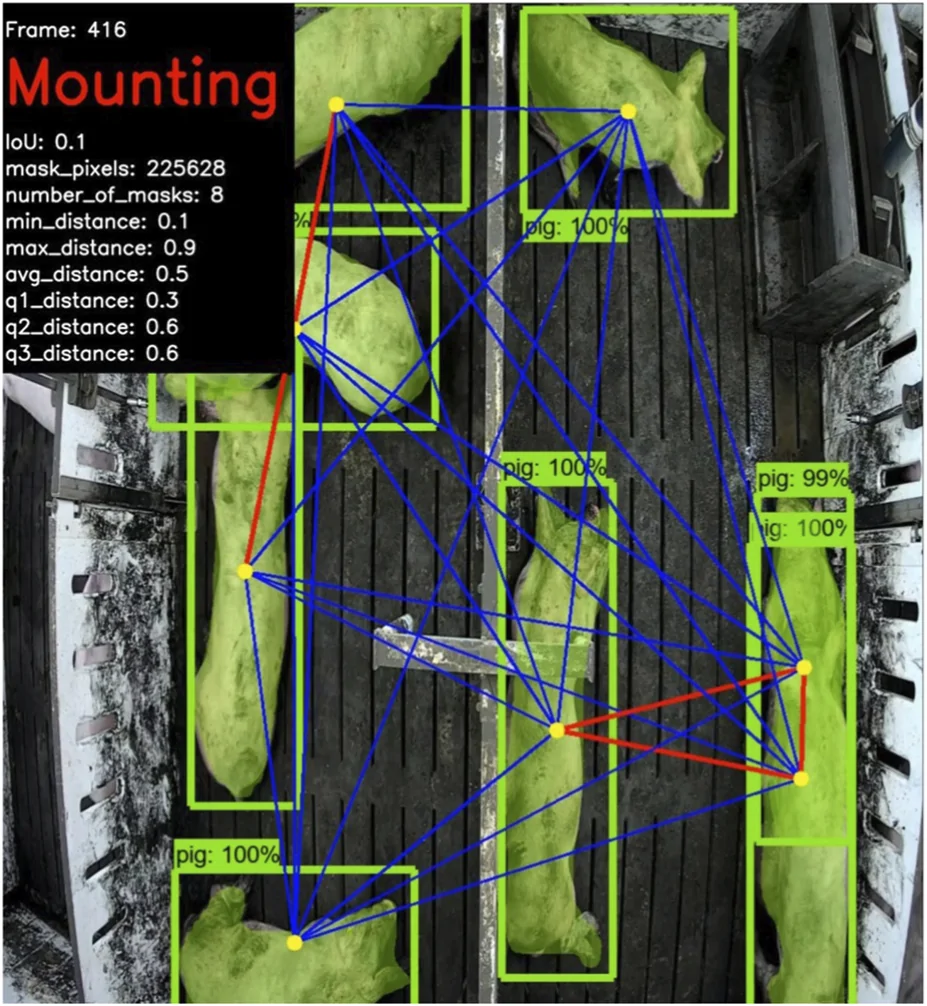
Combined model output of a video frame depicting mounting behaviour. A pig in the bottom right corner is mounting another pig, and the predicted behaviour label in the top right corner shows the correct classification of mounting, based on the input features shown below it, generated by the instance segmentation model.

Figure 8 shows the output of the hybrid model on a single video frame, showing the pigs in the pen, highlighted in green, detected by the instance segmentation model, and the current predicted behaviour label in the top left, generated by the binary classification model. Below the predicted behaviour label are the input features that the binary classification model uses. The red and blue lines denote shorter and longer distances between pigs, connected to their center points.

### 3.4 Computational efficiency

A key objective of this study was to develop an automated system capable of accurately and reliably detecting mounting behaviour in real-time. This is essential for assessing its practical applicability and determining whether it can be effectively deployed in production or research barn settings.

The system achieved an average processing speed of five frames per second, with each frame requiring approximately 0.2 s to progress from video input through instance segmentation to binary classification, ultimately producing a mounting or non-mounting prediction. This performance aligns with the benchmarks established by the developers of MASK-R-CNN, who also reported a processing speed of 5 FPS (He et al., 2020).

The hybrid model in this study maintains this efficiency by focusing on a single object class (pig) and applying a 100% confidence threshold for detection. Mounting detection was performed on individual frames without relying on continuous temporal tracking, allowing the system to process video streams in real-time, regardless of the input FPS, by analyzing one frame every 200 milliseconds and generating predictions at a rate of 5 FPS. Individual frame analysis also enables the possibility of skipping frames, reducing computing time. To analyze more pens simultaneously, simply assigning more computing resources to this task is a straightforward option, making the system scalable.

### 3.5 Comparison with previous studies

This study advances the understanding of how data grouping strategies impact ML model development, performance evaluation, and generalization. Grouping data into more distinct categories, such as by clip or day, enables models to learn generalizable behavioural patterns rather than data specific details. This leads to a more realistic assessment of model performance under practical conditions, particularly when applied to new or unseen data. Additionally, the proposed system employs a multi-feature approach that enhances detection robustness in the presence of common challenges such as occlusion and segmentation errors. By integrating IoU, mask-pixel count, and distance measures, the system compensates for imperfect detections and reliably identifies mounting behaviour in group-housed pigs.

One of the early studies using computer vision to detect mounting behaviour in pigs was conducted by (Nasirahmadi et al., 2016), who recorded overhead videos of mixed-sex growers weighing around 30 kg raised in groups of 22–23 animals. The researchers used ellipse fitting to localize pigs and calculated Euclidean distances between points or areas of interest such as head and tail, head and sides, and axis points to identify mounting events. A deep learning model known as Faster R-CNN (Faster Region-based Convolutional Neural Network) was employed to detect and locate pigs within each image (Ren et al., 2017). This model proposes potential object locations representing pigs, then classifies and refines these areas to achieve precise localization and identification. Their method attained a sensitivity of 94.5%, a specificity of 88.6%, and an accuracy of 92.7% for mounting detection using an approach based on individual frames. More recently, (Yang et al., 2021) also used Faster R-CNN to detect mounting behaviours in pigs using overhead video footage, focusing on estrus monitoring in sows. Frames were analyzed to extract features such as distance, overlap area, and intersection angle, which were input to an Extreme Gradient Boost (XGBoost) model to differentiate between mounting and non-mounting behaviours. This method enabled the development of a mounting detection model with an average accuracy of 95.2% when grouping data per clip.

Under frame-based data grouping, our hybrid system, using an SVM binary classifier, achieved a classification accuracy of 99%. In comparison, (Nasirahmadi et al., 2016) and (Li et al., 2019) reported classification accuracies of 92.7% and 91.5%, respectively, under similar conditions. When grouped by clips, our model achieved an accuracy of 95%, closely aligning with the 95.2% reported by (Yang et al., 2021). However, their method required pigs to be physically marked with numeric identifiers to track individuals involved in mounting, a strategy that poses challenges since markings can wear off or become obscured, leading to missed detections (Yang et al., 2021).

Importantly, our pilot study is the first to develop an automated system for detecting mounting behaviour in non-miniature, intact boars. Previous studies have focused primarily on castrated males, miniature male pigs (with unspecified castration status), and sows (Yang et al., 2021; Nasirahmadi et al., 2016; Li et al., 2019). Since mounting behaviour is more prevalent in boars and reflects individual temperament rather than random activity, the model presented here is well-positioned for follow-on evaluation in commercial boar facilities, conditional on multi-site validation across breeds, lighting conditions, and camera placements. Once validated at scale, this approach could support the identification and management of individual animals with elevated mounting tendencies, supporting welfare research and informing the design of targeted intervention strategies in future deployments (Lange et al., 2021; Hintze et al., 2013).

A growing body of work uses deep learning to detect mounting and related social behaviours across various livestock species. Two recent systematic reviews provide useful framing. (Rohan et al., 2024) reviewed deep-learning applications across cattle, pigs, sheep, goats, poultry, and horses, identifying pigs and cattle as the dominant species in livestock behaviour-recognition research. (Fazzari et al., 2025) surveyed audio, visual, and audiovisual deep-learning architectures for animal behaviour analysis and highlighted instance-segmentation-based interaction analysis as a relatively underexplored direction. Juhos et al. (2025) (Juhos et al., 2025) reviewed livestock aggression, including mounting in pigs, and noted that current pig-behaviour models tend to identify single behaviours within constrained contexts. Within this landscape, the closest methodological analogue to our work is (Noe et al., 2022), who combined Mask R-CNN instance segmentation with Kalman-filter tracking to detect estrus mounting in Japanese Black cattle on an outdoor ranch, achieving 95.5% detection accuracy and a segmentation F1 of 75.6% on roughly 2000 images. For dairy cattle in indoor housing, (Wang et al., 2023) reported a lightweight YOLOv5s detector for mounting recognition achieving mAP@0.5 of 0.877 on 3343 images. The present study extends this literature in three ways. First, we report the standard instance-segmentation measures expected in current computer-vision practice (mask AP50 = 91.9%, mask AP75 = 75.2%, mean mask IoU = 81.8%; Table 1). Second, we explicitly evaluate performance under three data-grouping protocols, by frame, by clip, and by day, exposing the gap between optimistic frame-based estimates and the more realistic clip- and day-based generalisation. Third, our hybrid Mask R-CNN + binary-classifier architecture is, to our knowledge, the first such system applied to non-miniature intact boars, a category for which mounting behaviour is both more prevalent and more behaviourally informative than in the castrates, miniature pigs, or sows examined in previous pig-mounting work.

## 4 Conclusion

This pilot study demonstrates the feasibility of an automated hybrid system that integrates Mask R-CNN instance segmentation with an SVM binary classifier to detect mounting behaviour in group-housed boars. Performance depended strongly on the data-grouping strategy. Under clip-based grouping, which represents the most realistic measure of within-recording generalisation, the system achieved a balanced accuracy of 95%. The day-based grouping, which holds out an entire day of recordings and represents the most challenging generalisation setting available in this pilot, yielded 77%, while the frame-based grouping yielded 99%. We report this number as an inflated upper bound arising from near-identical adjacent frames distributed across training and testing splits, and caution against interpreting it as deployment-level performance. These results highlight the critical impact of data organization on model performance and generalization capability evaluations.

A key strength of the hybrid system is its resilience to typical challenges in confined livestock environments, such as occlusions and variations in pig postures. By leveraging multiple features, including mask number, mask pixels, Intersection over Union (IoU), and inter-pig distances, the system maintains robust classification performance even when pig detections are imperfect. Furthermore, this multi-feature approach is compatible with standard 2D cameras, eliminating the need of expensive 3D imaging systems and facilitating future commercial-scale evaluation pending multi-site validation. Ultimately, this design supports precision livestock farming applications aimed at reducing stress, injury, and aggression related to mounting behaviour among group-housed pigs.

Beyond improving welfare and management in swine production, the study provides methodological insights into how different data grouping strategies influence machine learning outcomes. Grouping data by clips or days encourages models to learn underlying behavioural patterns rather than memorizing data-specific visual cues, leading to more realistic performance assessments of generalization to novel data. However, the results also emphasize the need for sufficient and diverse training data to support model generalization, particularly when data is grouped by days.

## 5 Limitations and future research

Despite the carefully designed experimental setup, this pilot study faced several limitations and revealed opportunities for future research. A key challenge was the presence of overlapping pigs due to non-mounting behaviours, such as stepping over, lying on top of, or sleeping beside one another, which can visually resemble mounting and lead to false positives. Including pose detection and body orientation analysis could help address this issue by providing additional distinguishing features, such as the direction the pigs are facing to better differentiate these behaviours.

Camera placement also introduced constraints. The reliance on a single camera overseeing two pens introduces perspective distortion, where pigs positioned farther from the center of the frame are more likely to be obscured by pen walls or other pigs. A potential improvement would be to mount cameras directly above each pen to enhance visibility. Mounting dedicated cameras above each pen rather than over pairs of pens would enhance visibility, though this configuration introduces additional infrastructure costs (camera units, cabling, computing) and maintenance demands (camera cleaning, occasional repositioning). While these increments are small relative to existing pen-side instrumentation and could be partially offset by reduced labour for direct behavioural observation, a formal cost-benefit analysis remains beyond the scope of this pilot. Furthermore, dataset constraints limit immediate commercial deployment. Because all recordings were captured under uniform conditions with the same pigs and pens, the model’s capacity to generalize to novel environments is restricted. The dataset also presents an inherent behavioral imbalance, as mounting behavior is primarily driven by a small subset of individuals, while the majority engage in it infrequently (Hintze et al., 2013). Consequently, external validation across diverse farms, breeds, lighting conditions, stocking densities, and camera placements is a prerequisite for commercial viability. This pilot is thus best understood as a proof-of-concept justifying multi-site follow-on studies.

To enhance adaptability, future deployments in novel settings should utilize partial retraining with smaller, site-specific datasets. Looking ahead, a promising avenue of research involves integrating scalable tracking technology to attribute detected behaviors to individual pigs for behavioral profiling. While (Yang et al., 2021) demonstrated individualized monitoring by marking pigs with numbered identifiers on their backs, their approach faces scalability challenges and suffers from issues such as fading, becoming obscured, or requiring frequent maintenance. Overcoming these tracking limitations will enable targeted interventions to improve animal welfare and productivity. Ultimately, with further development, the hybrid approach presented in this study could serve as an effective tool for automated behaviour monitoring in swine production, contributing to improved animal welfare, early detection of aggressive individuals, and more effective management in swine production systems.

## Data Availability

The raw data supporting the conclusions of this article will be made available by the authors, without undue reservation.
